# A 120‐year record of resilience to environmental change in brachiopods

**DOI:** 10.1111/gcb.14085

**Published:** 2018-03-14

**Authors:** Emma L. Cross, Elizabeth M. Harper, Lloyd S. Peck

**Affiliations:** ^1^ Department of Earth Sciences University of Cambridge Cambridge UK; ^2^ British Antarctic Survey Natural Environment Research Council Cambridge UK

**Keywords:** climate change, global warming, museum specimens, ocean acidification, shell characteristics

## Abstract

The inability of organisms to cope in changing environments poses a major threat to their survival. Rising carbon dioxide concentrations, recently exceeding 400 μatm, are rapidly warming and acidifying our oceans. Current understanding of organism responses to this environmental phenomenon is based mainly on relatively short‐ to medium‐term laboratory and field experiments, which cannot evaluate the potential for long‐term acclimation and adaptation, the processes identified as most important to confer resistance. Here, we present data from a novel approach that assesses responses over a centennial timescale showing remarkable resilience to change in a species predicted to be vulnerable. Utilising museum collections allows the assessment of how organisms have coped with past environmental change. It also provides a historical reference for future climate change responses. We evaluated a unique specimen collection of a single species of brachiopod (*Calloria inconspicua*) collected every decade from 1900 to 2014 from one sampling site. The majority of brachiopod shell characteristics remained unchanged over the past century. One response, however, appears to reinforce their shell by constructing narrower punctae (shell perforations) and laying down more shell. This study indicates one of the most calcium‐carbonate‐dependent species globally to be highly resilient to environmental change over the last 120 years and provides a new insight for how similar species might react and possibly adapt to future change.

## Graphical abstract

Current knowledge of how organisms will respond to ocean warming and acidification is based on relatively short‐ to medium‐term laboratory and field experiments, which cannot evaluate the potential for adaptation. We present a novel approach of utilising museum collections of a brachiopod species collected every decade at one site from 1900 to 2014 to assess how organisms have coped with past environmental change. This study indicates the resilience of one of the most vulnerable species globally to environmental change over the past 120 years and provides an insight for how similar species might react to future change.

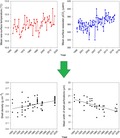

## 1 INTRODUCTION

Increased atmospheric carbon dioxide since the Industrial Revolution and subsequent rises in seawater temperature and decreases in pH have been well documented (Caldeira & Wickett, 2003, 2005; IPCC, 2013; Orr et al., 2005). Biological implications of these changes are less well described and have largely been identified from organism responses in laboratory experiments lasting a few days to a few months (Riebesell & Gattuso, 2015). In recognition of the fundamental role played by seasonal phenotypic plasticity and genetic change across generations, long‐term experiments which allow for acclimation (Cross, Peck, & Harper, 2015; Cross, Peck, Lamare, & Harper, 2016; Hazan, Wangensteen, & Fine, 2014; Suckling et al., 2014) and/or adaptation potential in organisms with short generation times (Andersson et al., 2015; Collins, Rost, & Rynearson, 2014) are now being made. Although information from long‐term laboratory experiments is vital to reveal sensitivities of marine organisms, even they can still only predict responses from exposures of relatively short durations, of months or even a few years, to environmentally unrealistic conditions (Andersson et al., 2015; Riebesell & Gattuso, 2015). Field experiments, including in situ mesocosms (Nagelkerken & Munday, 2015) and CO_2_ vent sites (Fabricius et al., 2011; Hall‐Spencer et al., 2008; Uthicke et al., 2016), are another common approach which allows for the investigation of impacts on more long‐term scales and also often include responses at the community level and the physical, chemical and biological variability in their natural environments that cannot be recreated in laboratory experiments. This method, however, has a lack of control of treatment conditions where organisms, for instance near vent sites, are locally exposed to significant short‐term variation in pH levels as well as vents releasing other harmful substances (Gattuso et al., 2014). The newest methods in ocean acidification research are the free‐ocean CO_2_ enrichment (FOCE) systems, which are designed to assess the impact of lowered pH on biological communities in situ over weeks to months (Gattuso et al., 2014). These systems include natural daily and seasonal pH changes as well as interspecific relationships and food webs (Barry et al., 2014; Kirkwood et al., 2015; Kline et al., 2012); however, the logistics of these systems are extremely challenging with replication a particular limitation due to cost and feasibility (Gattuso et al., 2014). A different and rarely used approach in the ocean acidification community is to evaluate changes over many decades in museum collections to determine how organisms have been affected by past environmental change. This provides a historical record of the effects of changing environments on marine organisms (Hoeksema et al., 2011; Lister, 2011), which complements widely used laboratory and field experiments by allowing the assessment of possible long‐term adaptation and presents a more holistic understanding of species responses.

Brachiopods are one of the best model groups of organisms to determine responses to climate change as they inhabit all oceans from intertidal to hadal depths (James et al., 1992; Peck, 2001) and are one of the most calcium‐carbonate‐dependent marine groups because their calcareous skeleton and other support structures usually make up >90%, and sometimes >95%, of their dry mass (Peck, 1993, 2008), values which are amongst the highest reported for any marine invertebrate to date. They have also been locally important for shallow and deep‐water communities for over 550 million years by providing a habitat for a diverse range of epifauna including encrusting sponges and algae (Barnes & Peck, 1996). A large‐scale loss of brachiopods would therefore not only affect local communities, but could also have wider consequences which potentially could lead to changes or imbalances in benthic ecosystems (Peck, 2008).

**Figure 1:**
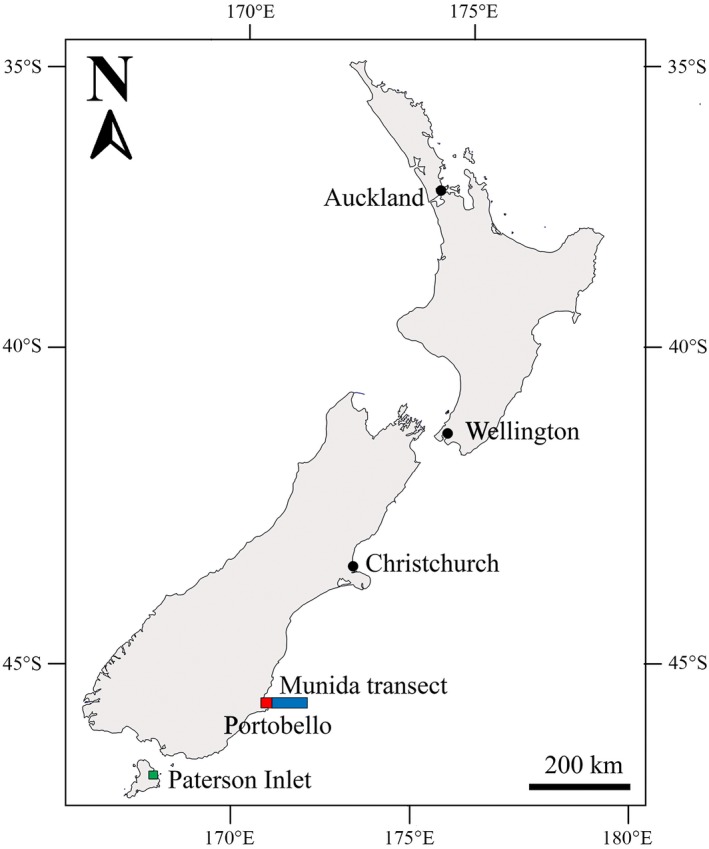
Location of specimen collection site (green square; Paterson Inlet, Stewart Island, New Zealand; 46.6°S, 168.1°E), surface seawater temperature site (red square; Portobello, Dunedin, New Zealand; 45.8°S, 170.7°E) and seawater *p*CO_2_ transect (blue line; off the coast of Dunedin, New Zealand; 45.8°S, 170.7°E to 45.8°S, 171.5°E) [Colour figure can be viewed at http://wileyonlinelibrary.com]

Brachiopods are common in all the world's oceans. They are, however, only abundant in a few areas and in New Zealand many species are highly abundant at relatively accessible shallow depths (<30 metres), and at several different locations (Rudwick, 1962). This includes *Calloria inconspicua* in Paterson Inlet, Stewart Island (Figure 1; Doherty, 1979) where marine biological exploration began in the early 1900s because of its high biodiversity (Willan, 1981). As a result, there are excellent museum collections of this species deposited at regular intervals over the last century from this site, making *C. inconspicua* an ideal species to investigate variation in shell characteristics since the Industrial Revolution. Environmental change in New Zealand waters over the last two decades is also in line with global trends of a 0.1 pH unit decrease and 2°C warmer (Bates et al., 2014; Law et al., 2017). The aims of this study, therefore, were to determine whether past environmental change had affected shell morphology, structure, elemental composition and integrity in museum specimens of *C. inconspicua* collected from a single site (Paterson Inlet) every decade from 1900 to 2014.

## 2 MATERIALS AND METHODS

### 2.1 Specimen and environmental data collection

In all, 389 specimens of *C. inconspicua* from every decade from 1900 to 2010 except the 1990′s from Paterson Inlet, Stewart Island, New Zealand (Figure 1; 46.6°S, 168.1°E) were evaluated from various museums and research institutions in New Zealand. In all, 15 individuals from 2014 were hand collected live from the sampling site. Non‐destructive morphometric measurements were made on specimens >16 mm in length as individuals become sexually mature at 14–16 mm length in *C. inconspicua* (Doherty, 1979). Only subtidal specimens >16 mm in length (maximum length: 25.5 mm) with detailed descriptions of location, date and depth of the sample collection site were used to eliminate depth and size bias to the analyses. Three to ten specimens from each decade were donated for further destructive shell analysis depending on the available museum collection sample size. Details on the specific location and depth of each sample are given in Table S1. Long‐term datasets of surface seawater temperatures were provided by Dr Doug Mackie and surface seawater *p*CO_2_ by Dr Kim Currie.

### 2.2 Shell characteristics

Eight key shell characteristics were analysed to determine any change over the past century. Shell morphology was assessed by measuring shell length, breadth and height of 389 individuals to the nearest 0.1 mm using Vernier calipers. A calcification index was calculated on 70 specimens (3–15 specimens per decade) that were donated for further shell analysis to quantify any variation in the efficiency of calcification. Calcification index was quantified as the amount of internal living space produced per unit of shell material deposited (Graus, 1974), therefore, calculated from the following equation:

Calcification index = dry weight of the shell (g)/internal volume of the shell (cm^3^)

Dry weight was measured to 0.001 g on a Sartorius LA3200D weighing balance and the volume measurements were made according to Peck (1992).

To investigate any changes in shell structure, shell density, punctal (shell perforations) density and punctal width were measured. Shell density was calculated for the same 70 individuals used in the calcification index analysis using the following equation:

Shell density = dry weight of the shell (g)/shell volume (cm^3^)

Punctal densities (mm^−2^) were calculated from Scanning Electron Microscope (SEM; FEI QEMSCAN 650F) micrographs (1 mm^2^) of the outer surfaces of 40 pedicle valves (2–5 valves per decade) from 10 different areas on each specimen (Figure S1). Punctae are spatially distributed in terebratulids at regular intervals of ~45 μm in a dominantly hexagonal, close packing pattern throughout each valve with minimal spatial variability (±5 punctae per mm^2^) (Williams, 1997). Punctal widths were measured from acetate peels (prepared according to Richardson, Crisp, and Runham (1979)) of cross sections of 40 brachial valves (2–5 valves per decade). Ten punctae were measured per specimen across the length of the individual to the nearest 0.1 mm on a Swift monocular petrological microscope with fitted micrometer. The percentage of shell that is punctae vs. shell matrix was then calculated from firstly calculating the mean area of a punctum for each year, where area is calculated as π*r* ^2^ and r is half the mean punctal width.

The mean area of a punctum (mm^2^) and the mean punctal density of 152 mm^−2^ were then used to calculate the percentage area of shell occupied by punctae for each year by the following equation:

Percentage area of shell occupied by punctae = (mean area of punctum x mean punctal density) × 100

The percentage change in shell occupied by punctae compared to shell matrix was calculated by solving the linear regression equation relating shell area occupied by punctae for 1900 and 2014.

Solubility of skeletal structures is partly controlled by the elemental composition of the calcite crystal lattice (Harper, 2000; LaVigne et al., 2013). Any ion substitutions through changes in environmental variables, such as temperature (Chave, 1954), seawater composition (Ries, 2010) and seawater saturation state (Ries, 2011), could increase mineral solubility (LaVigne et al., 2013; Morse, Arvidson, & Lüttge, 2007). Elemental composition of the shell was, therefore, analysed using a Cameca SX100 electron microprobe operated at 15 keV acceleration voltage, a 20 nA beam current and a 5 μm spot size. Three vertical profiles from the outer surface of the shell towards the inner surface were measured near the umbo, in the middle of the shell and near the shell margin from cross sections of 20 brachial valves (3 valves per 20 years). Barium (Ba), calcium (Ca), iron (Fe), magnesium (Mg), manganese (Mn), phosphorus (P), silicon (Si), sodium (Na) and strontium (Sr) were chosen to be measured for comparison to previous ocean acidification studies assessing effects on elemental composition of calcium‐carbonate‐dependent organisms (Bray, Pancucci‐Papadopoulou, & Hall‐Spencer, 2014; Hahn et al., 2012; LaVigne et al., 2013). Apatite (P), benitoite (Ba), celest (Sr), diopside (Ca & Si), fayalite (Fe), jadeite (Na), manganese (Mn) and olivine (St John's) (Mg) were used as standards. All minor elements (Si, Fe, Mn and Ba) were below detection limits and, therefore, excluded from further analysis. Matrix correction was performed following Pouchou and Pichoir (1984) (the PAP procedure). Standard analysis reproducibility was <1% for each element analysed. PAP corrected data were stoichiometrically calculated as carbonate (Reed, 1993).

To investigate any changes in shell integrity, a shell condition index and shell thickness were measured. Shell condition index was determined through measuring percentage areas of four types of shell condition (Table S2) in ImageJ from 1 mm^2^ SEM micrographs of the outer shell surfaces of 40 pedicle valves (2–5 valves per decade) at 10 different areas on each specimen (Figure S1). Primary layer, secondary layer and total shell thickness measurements (±0.1 mm) were made from acetate peels of cross sections of 40 brachial valves (2–5 valves per decade) near the umbo, in the middle of the shell and near the shell margin on each specimen on a Swift monocular petrological microscope with fitted micrometer.

### 2.3 Statistical analyses

Calcification index, shell density, punctal density, punctal width and shell thickness data were all normally distributed (Anderson–Darling test; *p* > .05). Parametric linear regression analyses were, therefore, performed on these characteristics to determine whether they changed over the last 120 years. The regression models for the calcification index and shell density datasets included year as the only factor as all measurements were made on different individuals. As punctal density, punctal width and shell thickness measurements were conducted at several points within an individual, their regression models also included individual number as a random effect with year as the fixed effect. Overall morphometric and elemental composition raw data were both non‐normally distributed even after square root, log and arcsine transformations. Log‐transformed data in each year for each morphometric measurement were, however, normally distributed (Anderson–Darling test; *p* > .05). Therefore, parametric multiple regression analyses were conducted on each morphometric measurement to determine whether each relationship with size varied over time. Similarly, raw data in each individual year for Ca, Na and Sr and log‐transformed data for each individual year for Mg and P were normally distributed (Anderson–Darling test; *p* > .05). As multiple measurements were analysed within an individual, parametric linear regression analyses with year as the fixed effect and individual number as the random effect were performed on each element to determine whether elemental composition changed over the last 120 years. Shell condition index data (% area) were arcsine transformed to remove imposed limits, but the transformed data were not normally distributed because of zeros in the dataset (Anderson–Darling test; *p* < .05). Non‐parametric Kruskal–Wallis tests were, therefore, used to determine whether treatment affected the proportion of each shell condition. When there were significant differences, a further Kruskal–Wallis Multiple Comparisons test was used to identify differences between treatments. Statistical analyses were conducted with minitab (Statistical Software™ Version 17).

## 3 RESULTS

### 3.1 Environmental conditions

**Figure 2:**
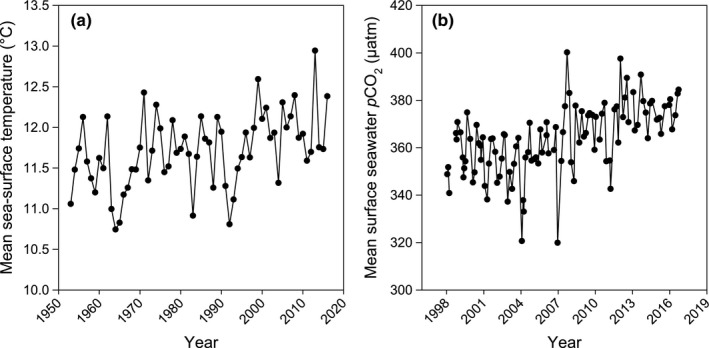
Long‐term monitoring of environmental change in southeast New Zealand: Annual mean sea‐surface temperature data from 1953 to 2016 from Portobello, Dunedin, New Zealand (dataset provided by Dr Doug Mackie) (a). Mean surface seawater *p*CO_2_ data from 1998 to 2016 measured 5–7 times per year from the Munida‐transect extending 65 km from Taiaroa Head at the entrance to Otago Harbour, Dunedin, New Zealand (dataset provided by Dr Kim Currie) (b)

Long‐term monitoring of environmental conditions over the last 20–60 years revealed significant increases in sea‐surface temperature (SST; Linear Regression: *R* ^2^ = .19, *F*_1,63_ = 14.22, *p* < .000) and surface seawater partial pressure of CO_2_ (*p*CO_2_; Linear Regression: *R* ^2^ = 0.31, *F*_1,104_ = 45.60, *p* < .000). SST rose by 0.6°C from 1953 to 2016 in Portobello, Dunedin, New Zealand (Figure 2a) and *p*CO_2_ increased by 35.7 μatm from 1998 to 2016 off the coast of Dunedin, New Zealand (Figure 2b).

### 3.2 Shell characteristics

**Figure 3:**
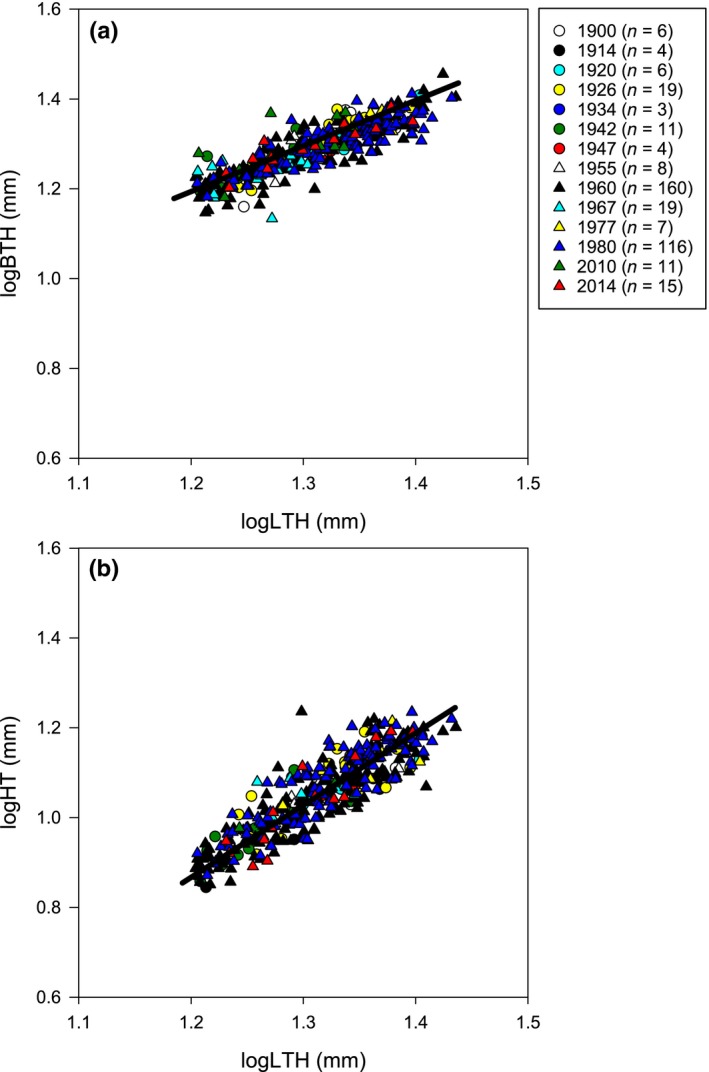
Relationships between breadth (a) and height (b) with length over the last 120 years. Each different symbol represents a different year (see legend). Regression lines are plotted for all specimens

Non‐destructive morphometric measurements of length, breadth and height in individuals >16 mm in length revealed that breadth increased almost proportionally to length with size (slope =0.90), but height increased more than length (slope=1.44) with size (Figure 3; Table S3). Growth is, therefore, not isometric and shells get taller as they grow. The relationships of length to breadth and length to height did not change over time (Figure 3; Multiple regression analysis LTH/BTH: *F*_13,386_ = 1.33, *p* = .193; LTH/HT: *F*_13,364 _= 1.15, *p* = .315).

**Figure 4:**
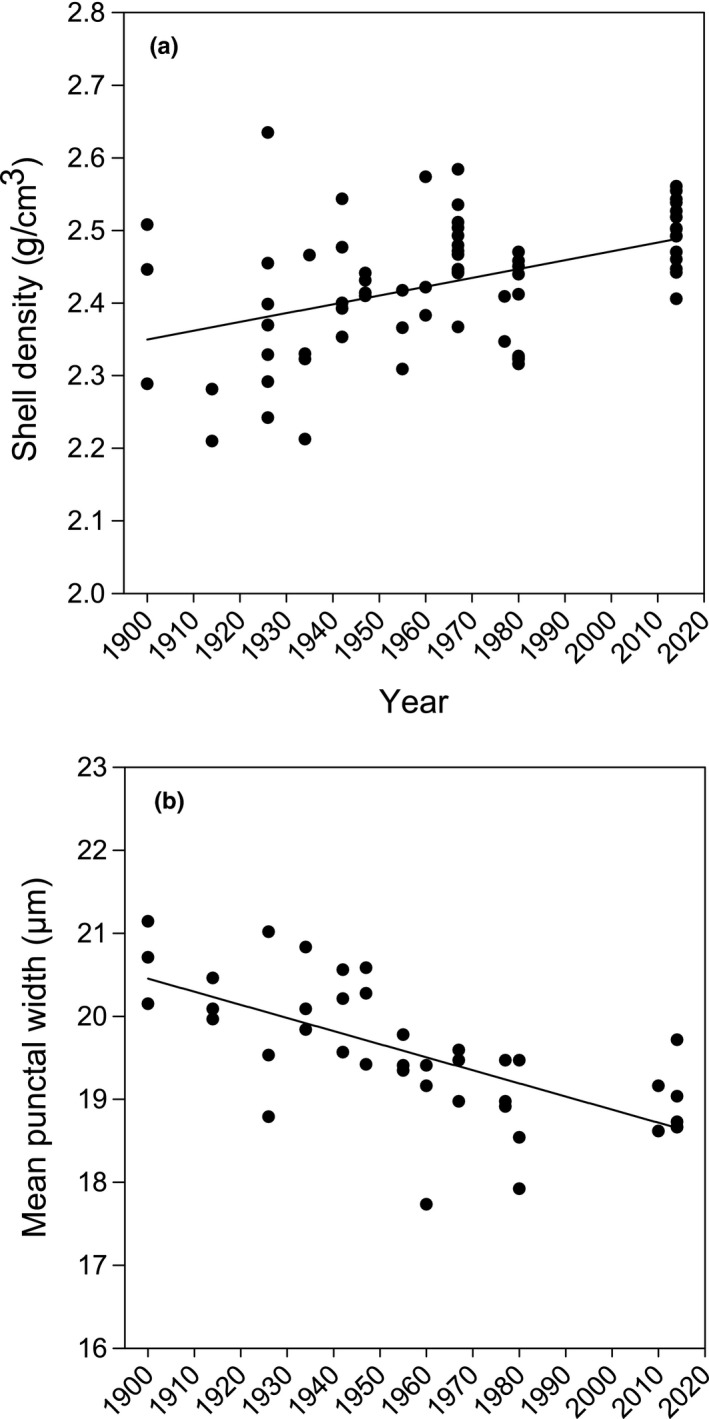
Shell density increase (a) and punctal width decrease (b) over the last 120 years. Error bars removed for clarity

**Figure 5:**
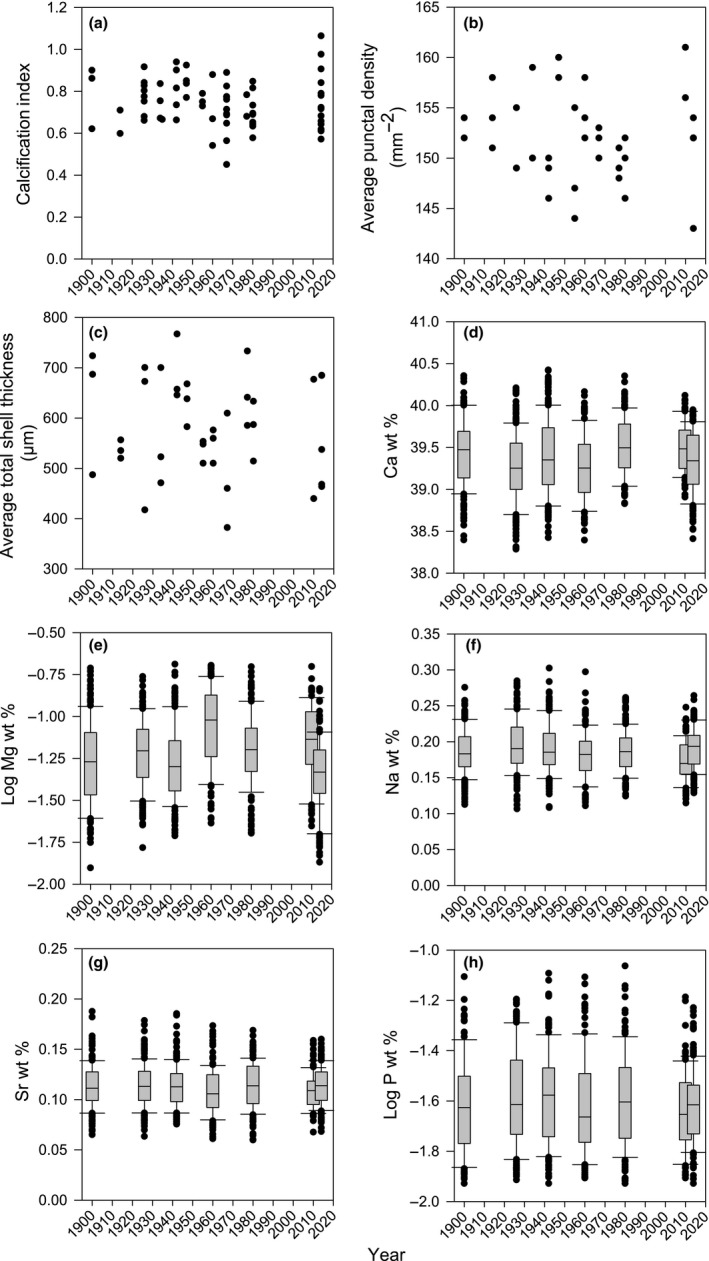
Key shell characteristics, including calcification index (a), punctal density (b), total shell thickness (c) and elemental composition (d–h) that have not varied in *Calloria inconspicua* over the last 120 years. Error bars removed for clarity in a–c only

Further destructive analysis of seven key shell characteristics revealed that shell density increased by 3.43% from 1900 to 2014 (Table 1; Figure 4a) which cannot be explained by changes in the ratio of shell mass to volume enclosed by the shell (calcification index; Table 1; Figure 5a), by the density of shell perforations (punctae; Table 1; Figure 5b), or by total shell thickness (Table 1; Figure 5c) as none of these characteristics varied over this time period. Shell elemental composition for the main five components (Ca, Mg, Na, Sr and P) also did not change over the last 120 years (Table 1; Figure 5d–h). Punctal width, however, decreased by 8.26% (Table 1; Figure 4b). This equated to a 1% decrease in shell occupied by punctae, which explains part of the 3.43% increase in shell density.

**Table 1:** Statistical results from parametric linear regression (using *R* ^2^ and *F* statistic) and non‐parametric Kruskal–Wallis (using *H* statistic) analyses of each shell characteristic over the last 120 years. *p* values in bold denote statistically significant difference

Shell characteristic		*R* ^2^	Factors	Degrees of freedom	*F* or H*	*p* (<.05)
Shell morphology	Calcification index	.009	Year	1, 68	0.63	.429
Shell structure	Shell density	.200	Year	1, 69	17.16	**<.001**
	Punctal density	.017	Year	1, 38	0.21	.648
			Individual number	2, 38	0.25	.779
			Year*individual number	2, 38	0.25	.781
	Punctal width	.456	Year	1, 38	5.13	**.030**
			Individual number	2, 38	0.38	.685
			Year*individual number	2, 38	0.39	.680
Shell integrity	Dissolution	‐	Year	‐	19.37*	.080
	Total shell thickness	.117	Year	1, 37	0.74	.396
			Individual number	2, 37	1.23	.304
			Year*individual number	2, 37	1.22	.308
Shell elemental composition	Ca concentration	.100	Year	1, 14	0.09	.766
			Individual number	2, 14	0.16	.851
			Year*individual number	2, 14	0.01	.990
	Mg concentration	.134	Year	1, 14	1.09	.315
			Individual number	2, 14	0.76	.485
			Year*individual number	2, 14	1.06	.373
	Na concentration	.324	Year	1, 14	0.27	.612
			Individual number	2, 14	0.30	.742
			Year*Individual Number	2, 14	0.11	.899
	Sr concentration	.300	Year	1, 14	0.04	.844
			Individual number	2, 14	0.70	.514
			Year*individual number	2, 14	1.93	.181
	P concentration	.100	Year	1, 14	0.99	.321
			Individual number	2, 14	1.89	.152
			Year*individual number	2, 14	1.92	.103

**Figure 6:**
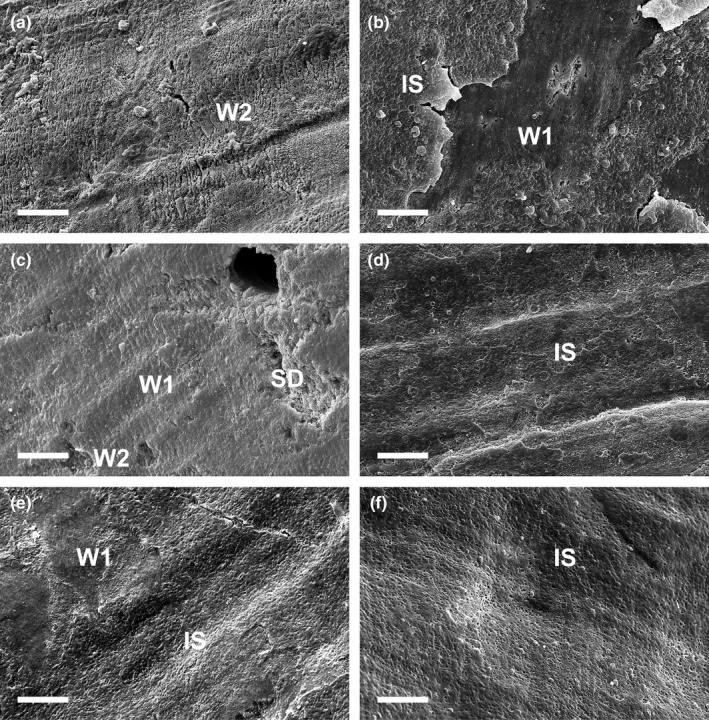
Examples of scanning electron microscope (SEM) micrographs of shell surfaces every 20 years over the last 120 years including from 1900 (a), 1926 (b), 1947 (c), 1967 (d), 1980 (e) and 2014 (f). IS, intact periostracum with pitted layer, W1, intact periostracum without pitted layer (minimal wear), W2, wear but no shell dissolution (extensive wear) and SD, shell dissolution. Scale bar = 20 μm

The majority of shell surfaces (>55% in each decade) of specimens throughout the 120‐year study were intact with the protective periostracum layer undamaged and with the outer pitted layer present (Figure S2). Only minimal shell dissolution (0%–13%) occurred in any specimen, and this did not vary throughout the time series (Figure 6.; Table 1).

## 4 DISCUSSION

Long‐term monitoring of environmental conditions in the southeast of New Zealand over the last 20–60 years (Figure 2) are in line with global trends of our oceans becoming 2°C warmer and 0.1 pH units more acidic since the Industrial Revolution (Caldeira & Wickett, 2003, 2005; IPCC, 2013; Orr et al., 2005). The resilience of *C. inconspicua* to environmental change over the last century is clear from the data on various shell characteristics in this study. Six key aspects of the shells of this high calcium carbonate content species did not change since 1900 through to 2014 despite significant environmental shifts of a 0.6°C SST increase over the last 60 years and 35.7 μatm increase in surface seawater *p*CO_2_ over the last 20 years.

Morphometrics in this study, as expected, revealed that shells get taller as they grow, providing more space for larger gonads and larval brooding, particularly after individuals become sexually mature (14–16 mm length; Rickwood, 1977; Doherty, 1979). Variance in shell shape is a result of space constraints as larvae settle on or next to older individuals forming dense conspecific clusters. Morphometric relationships did not differ over the last 120 years, which is in contrast to other shell‐bearing organisms in laboratory experiments where temperature and *p*CO_2_ have been reported to impact phenotypic plasticity and alter shell morphology (Fitzer et al., 2015; Peyer, Hermanson, & Lee, 2010). Temperature had the greatest effect on shell morphology in the mussel *Dreissena polymorpha* in comparison to food quantity and water motion (Peyer et al., 2010). Higher temperatures (~18 to 20°C) caused more rounded shells to be produced, whereas lower temperatures (~6 to 8°C) caused more laterally flattened shells. Increased *p*CO_2_ conditions (750 μatm and 1,000 μatm) resulted in rounder and flatter *Mytilus edulis* shells which also had a thinner aragonite layer compared to ambient conditions (380 μatm) (Fitzer et al., 2015). This new shell shape was explained as a compensatory mechanism to enhance protection from predators and changing environments due to the inability of this species to produce thicker shells under increased ocean acidity. The lack of a change in shell morphology of *C. inconspicua* over the last 120 years demonstrates the tolerance of this species to altered abiotic conditions.

Shell density increased by 3.43% from 1900 to 2014, which cannot be explained by a change in shell morphology, elemental composition, shell thickness or the number of punctae as none of these shell characteristics varied over this period. This demonstrates the robust control of several aspects of shell production in *C. inconspicua* to changing environmental conditions. Punctal width, however, significantly decreased by 8.26% demonstrating that this species appears to have laid down more shell by constructing narrower punctae. This response may increase protection from their changing habitat or predation pressure by reinforcing the shell structure. Low shell repair frequencies, however, have been observed in *C. inconspicua* from Paterson Inlet [Harper, pers. obs.]. This response is, therefore, unlikely a result of any changes in their predator populations over the last 120 years. Producing narrower punctae is more likely a response to acidification by increasing calcification as seen in some species (Wood, Spicer, & Widdicombe, 2008). Producing narrower punctae, however, could have physiological implications for rhynchonelliform brachiopods as there is less space for extensions of soft tissue, called caeca, from the mantle into the punctae (Peck & Holmes, 1989a). As the function of punctae is still under debate (Pérez‐Huerta et al., 2009), the extent, if any, of the impact on the organism remains unknown.

The majority of the shell surfaces throughout the 120‐year time period remained intact with the protective periostracum layer undamaged, the pitted layer present and only low levels of dissolution. The increase in seawater *p*CO_2_ since 1900, therefore, did not cause extensive dissolution or impact the outer protective periostracum. This is in contrast to studies investigating the effects of end‐century acidified conditions of a further increase to 1,000–1,300 ppm (IPCC, 2013) where dissolution is common amongst marine calcifiers including corals (Comeau, Carpenter, Lantz, & Edmunds, 2014), echinoderms (Dubois, 2014) and molluscs (Nienhuis, Palmer, & Harley, 2010). These rhynchonelliform brachiopods have therefore been unaffected in their abilities to construct and maintain their extensive skeletons by the change in ocean acidity and temperature over the last 120 years. Future conditions, however, could pose a threat to shell integrity and major questions around how far conditions need to change before significant impacts are evident remain to be answered.

Historical studies provide a different perspective to outstanding questions of how environmental change will impact marine life. Organism responses to past change provide valuable historic baselines on which we can test predictions of future biological impacts from commonly used laboratory and field analyses. The historical data we present here complement a 3‐month laboratory study demonstrating the strong resilience of shell growth and repair in *C. inconspicua* to future environmental change (Cross et al., 2016). Similar to other environmental change research methods, there are some limitations to utilising museum collections. These include unfocussed collecting bias towards larger individuals and a lack of specific collection details prohibiting the use of all available specimens. Such studies also rely on regular accurate environmental and specimen sampling from the same site over long‐term timescales. Despite being rarely used in ocean acidification and warming research, we believe this novel approach provides a unique way of evaluating possible long‐term adaptation. Varying responses of marine calcifiers over a wide range of timescales have been demonstrated in the few other historical studies and these have provided a new insight on the respective work into future environmental change impacts on the same species. Modern shells of *Mytilus californianus* from 2009 to 2011 and 1960s to 1970s were reported to be thinner than shells from Native American Midden sites dating back to ~1000 to 2420 years BP (Pfister et al., 2016). There was no decline, however, in adult shell thickness over the last 40 years. This is in contrast to larvae of *M. californianus* which produced thinner, weaker and smaller shells cultured under CO_2_ conditions predicted for 2,100 (970 ppm) than larvae raised under present‐day conditions (380 ppm) (Gaylord et al., 2011). Larvae, however, are often thought to be the most sensitive life stage to environmental change (Byrne, 2011). Calcification has also been reported to decline by 14.2% since 1990 in 328 colonies of the reef‐building coral *Porites* from 69 reefs of the Great Barrier Reef (De'ath, Lough, & Fabricius, 2009). Similarly, calcification was reported to decline by ~40% in *Porites* under high CO_2_ conditions (1,000–1,300 ppm) predicted for 2100 during an 8‐week laboratory experiment (Anthony, Kline, Diaz‐Pulido, Dove, & Hoegh‐Guldberg, 2008).

The current study is the first to present data of a wide range of shell characteristics of a potentially vulnerable species from a single site every decade since 1900 to 2014, which covers the second half of the post‐industrial revolution, when alterations in *p*CO_2_ have been strongest. Six out of eight key shell characteristics measured in this unique collection were not affected by environmental change. The only change observed was a decrease in punctal width which partially explained an observed increase in shell density, that may reinforce the structure of the shell. This indicates this highly calcium‐carbonate‐dependent species has been highly resilient to past changes in environmental conditions over the last 120 years and provides a novel insight into how similar species might react and adapt to future change.
