# Supplementary material for: A 120‐year record of resilience to environmental change in brachiopods
Source: Glob Chang Biol. 2018 Mar 14;24(6):2262–71. doi: 10.1111/gcb.14085 (PMC6850138; doi:10.1111/gcb.14085)
Supplement: Supplementary file 1 [file GCB-24-2262-s001.docx]

**Supporting Information**

Table S1. Specific details about the collection and storage sites of the museum specimens.

| Year | Sample ID | Institute sample obtained from | Specific location collected | Depth collected (metres) |
| --- | --- | --- | --- | --- |
| 1900 | BR000088 | Te Papa Museum | Stewart Island | Not given |
| 1914 | BR001686 |  | Oyster beds 3.2 km east of Halfmoon Bay, Stewart Island | 42 m |
| 1920 | BR.000058 |  | Foveaux Strait | Not given |
| 1926 | BR001062 |  | Golden Bay, Stewart Island (46° 54'S, 168° 7'E) | Not given |
| 1934 | 2006.12.140 | Canterbury Museum | Horseshoe Bay, Butterfly Beach, Stewart Island | Not given |
| 1942 | MA79298 | Auckland Museum | Halfmoon Bay, Stewart Island (46° 54'S, 168° 8'E) | Not given |
| 1942 | 2005.188.39 | Canterbury Museum | Oyster beds in Foveaux Strait | Not given |
| 1947 | AU20001 | University of Auckland | Foveaux Strait | 60 m |
| 1955 | BR001331 | Te Papa Museum | Halfmoon Bay, Stewart Island (46° 54'S, 168° 7'E) | Not given |
| 1960 | NIWA 62858 B243 | National Institute of Water and Atmospheric Research | Near Abrahams Bay, further in Paterson Inlet than Ulva Island, Stewart Island (46° 94'S, 168° 05'E) | 21 m |
| 1967 | NIWA 62892 E833 |  | In between Ulva Island and Big Glory Bay, Paterson Inlet, Stewart Island (46° 95'S, 168° 15'E) | 53 m |
| 1977 | NIWA 62891 K989 |  | East Ulva Island, Stewart Island (46° 94'S, 168° 16'E) | 22 m |
| 1980 | NIWA 62890 S265 |  | Deep Bay, North of Ulva Island, Paterson Inlet, Stewart Island (46° 91'S, 168° 12'E) | 10 m |
|  | NIWA 62919 S263 |  | East Ulva Island, Paterson Inlet, Stewart Island (46° 94'S, 168° 16'E) | 27 m |
| 2010 | Cruise OS15 | Department of Geology,  University of Otago | Just outside of Paterson Inlet (46° 59'S, 168° 16'E) | 42 m |
| 2014 | 2014 | Collected by Dr Miles Lamare | Groper Island, south of Ulva Island, Paterson Inlet, Stewart Island (46° 57’S, 168° 09’E) | 20 m |

**
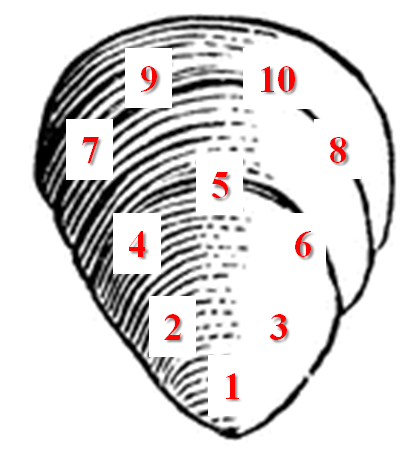
**

Fig. S1. A schematic of a pedicle valve indicating the 10 areas where Scanning Electron Microscopy micrographs were collected for punctal density and shell condition index analysis.

Table S2. Descriptions of each type of shell condition with SEM examples for shell condition index analysis. Scale bar = 10 µm.

| Shell condition index | Description | Scanning Electron Microscopy micrograph examples |
| --- | --- | --- |
| **Intact shell (IS)**:  Intact periostracum with pitted layer | Smooth, intact periostracum with no signs of wear or dissolution with a complete covering of the pitted layer on the surface. | 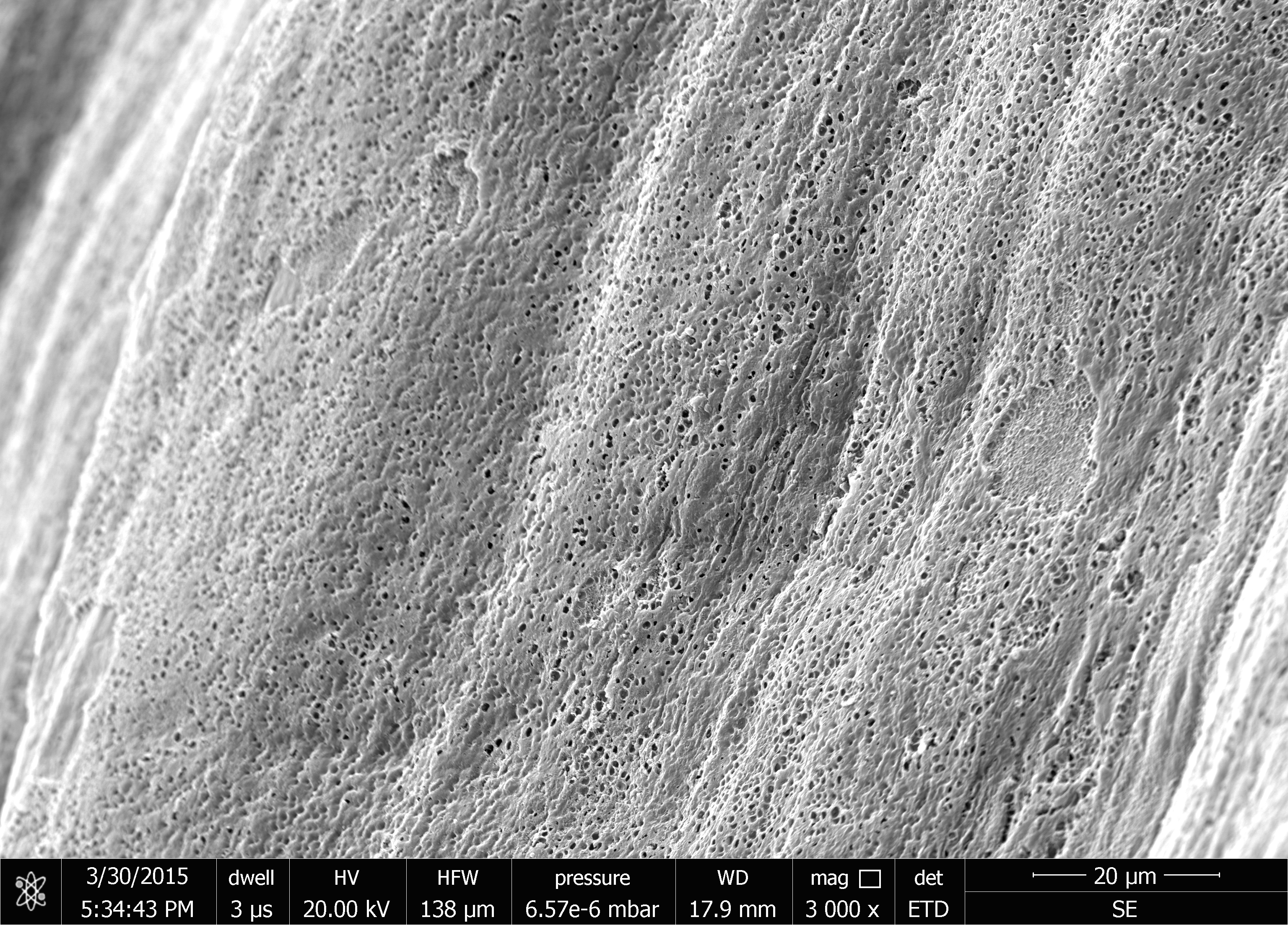 |
| **Minimal wear (W1)**:  Intact periostracum without pitted layer | Smooth intact periostracum without any extensive signs of wear, however, the pitted layer is absent probably due to less invasive wear. |  |
| **Extensive wear (W2)**:  Wear but no dissolution | Abrasion of the periostracum causes rough, blunt and sharp surfaces or cylindrical, hollow meandering paths from shell-boring organisms. Despite exposing the inner shell layers, no dissolution occurs. |  |
| **Shell dissolution (DS)**: Dissolution in the primary layer | Corrosion of the primary layer with flaky, dissolved calcite crystals. |  |

Table S3. Regression analysis coefficients on the size and shape of all 386 shells measured. *b* = slope, SE = standard error, SEy = standard error about the regression line.

| Independent (x) | Dependent (y) | *b* | SE*b* | Intercept | SEy | R^2^ |
| --- | --- | --- | --- | --- | --- | --- |
| logLTH | logBTH | 0.90 | 0.026 | 0.11 | 0.03 | 0.75 |
| logLTH | logHT | 1.45 | 0.042 | -0.85 | 0.04 | 0.77 |


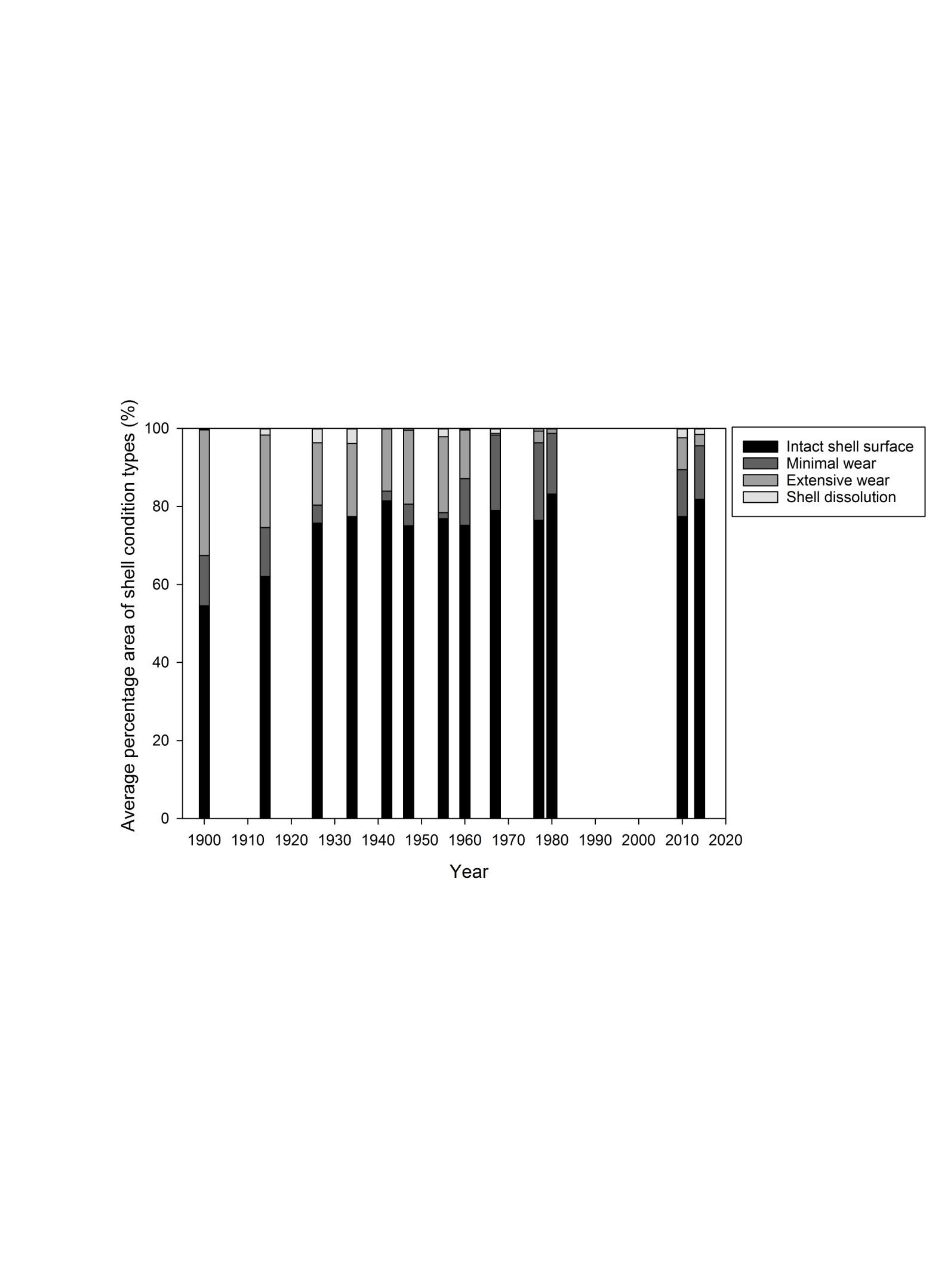


Fig. S2. Mean percentage area of the different types of shell conditions over the last 120 years. Lighter grey tones indicate an increase in wear and/or shell dissolution (see legend).
